# Are older teens more frustrated than younger teens by the covid-19 restrictions? The role of psychological maturity, personality traits, depression and life satisfaction

**DOI:** 10.1007/s12144-023-04317-6

**Published:** 2023-02-02

**Authors:** Fabia Morales-Vives, Pere J. Ferrando, Jorge-M. Dueñas, Sergi Martín-Arbós, M. Dolores Varea, Elena Castarlenas

**Affiliations:** 1 Psychology Department, Universitat Rovira i Virgili, Tarragona, Spain; 2 Research Center for Behavior Assessment (CRAMC), Tarragona, Spain; 3 Pedagogy Department, Universitat Rovira i Virgili, Tarragona, Spain

**Keywords:** COVID-19, Frustration, Maturity, Personality, Depressive symptoms, Life satisfaction

## Abstract

Many studies have shown the negative impact of lockdowns on adolescents, but there is less evidence on how they are affected by other stages of the COVID-19 pandemic, and, especially, whether there are any differences between early and late adolescence. The current study focuses on the frustration felt by adolescents because of the severe COVID-19 restrictions in a non-lockdown situation. We aimed to (a) assess the role of maturity and two personality traits (emotional stability and extraversion) in predicting their frustration, and (b) compare the levels of frustration, depressive symptoms, and life satisfaction in older and younger adolescents. The sample of older adolescents was also compared with a paired sample of the same age collected in 2018, before the pandemic. The results suggest that maturity, extraversion and emotional stability are predictors of frustration in both older and younger adolescents, although older adolescents reported higher levels of frustration and depressive symptoms, and lower levels of life satisfaction. Older adolescents also reported higher levels of depressive symptoms than adolescents of the same age before the pandemic. These results show the negative impact of the pandemic, especially on older adolescents, and the important role of maturity and some personality traits in predicting their frustration.

## Introduction

After the outbreak of the COVID-19 pandemic, governments around the world took preventive measures to stop the new coronavirus from spreading, to protect their citizens and to avoid the collapse of the healthcare services. On 14 March 2020, the Spanish government decreed a domestic lockdown that lasted about three months. All cultural and sporting events were cancelled, educational institutions at all levels closed, and non-essential businesses (i.e. everything except food shops and chemists) shut down, so all non-essential service workers had to stay at home. From 28 April 2020 onwards there was a progressive de-escalation and the situation became somewhat more flexible. Similar situations have occurred in different parts of the world, during the different stages of the pandemic, with lockdowns in some cases and other kind of measures and restrictions in others.

Although studies show that lockdown is an effective measure for controlling COVID-19 outbreaks (e.g., Lau et al., 2020; Nussbaumer-Streit et al., 2020), it can be an unpleasant experience because of the isolation and lack of contact with loved ones, especially for young people and adolescents (Morales-Vives et al., 2020; Panda et al., 2020). In fact, lockdown considerably restricts the possibilities of socialization with the peer group, which is particularly important for adolescents as it is a source of emotional and social support, and related to well-being, social development and long-term adjustment (Ellis & Zarbatany, 2017). But it is not only lockdowns that limit the social interaction among adolescents and young people during the pandemic. In other phases of the pandemic, restrictions and security measures put in place by governments have also limited the possibility of social interaction with others outside the nuclear family. In Spain, some of these measures were the closure of bars, restaurants, night clubs and other places frequented by young people in their leisure time, as well as curfews that did not allow people to go out at night, and restrictions on mobility that prevented people from leaving their own town or district. In adolescents, this lack of physical social interactions has been replaced to some extent by social media (Ellis et al., 2020), but the quality of these interactions, and the associated experiences, are not comparable to direct contact with the peer group. In fact, several studies show that increases in screen time are related to increases in depressive symptoms in adolescents (e.g., Boers et al.,, 2019; Maras et al., 2015).

## Adolescent’s mental health during the pandemic

The review by Loades et al. (2020) of articles published between 1946 and March 2020 provides considerable evidence of the negative impact of social isolation and loneliness on the mental health of children and adolescents. For this reason, these authors warned that the pandemic would have a negative impact on adolescents, whose levels of anxiety and depressive symptomatology would increase. In response, this would require preventive effort and early intervention. So, it is only to be expected that the pandemic has been reported to have had a negative psychological impact on adolescents, with an increase in anxiety, panic, depressive symptoms, somatizations, and non-suicidal self-injurious behaviours (e.g., Hawes et al., 2021; Kurudirek et al., 2022; Orgilés et al., 2021; Sultana et al., 2021; Tang et al., 2021). This deterioration in mental health has been observed in both community and clinical samples (Hawke et al., 2020). While most of these studies have been carried out during lockdown periods, there is also evidence to suggest that other phases of the pandemic generate worrying levels of anxiety and depressive symptomatology (e.g., Duan et al., 2022; Hajek et al., 2022).

## Frustration in adolescents during the most restrictive phases of the pandemic

The pandemic has given rise to psychological distress and concern among adolescents for numerous reasons. Restricted social interactions have been an important source of distress (Magson et al., 2021), but so has the concern about their own and others’ health, their family's economic situation, and their studies (Muñoz-Fernández & Rodríguez-Meirinhos, 2021). Furthermore, people may feel frustrated when they have to cope with rules and situations that they perceive as a barrier to their freedom and needs (Vansteenkiste & Ryan, 2013). Therefore, the preventive measures and restrictions linked to the pandemic can lead to frustration, which in turn may be another source of psychological distress. However, very few studies have focused on the frustration that adolescents may feel as a result of the restrictions associated with the pandemic. The study by Muñoz-Fernández and Rodríguez-Meirinhos (2021) showed the moderate to high levels of frustration that adolescents felt during the lockdown in Spain. This frustration was higher in girls than in boys, and was associated with the fact that they spent more time on online peer activities and had more COVID-19 related concerns. In contrast, higher levels of optimism, doing more leisure activities, and maintaining daily routines were associated with lower levels of frustration. In a qualitative, interview-based study, Gittings et al. (2021) also reported frustration and anxiety about the uncertainty of the future in South African adolescents during lockdown, with some adolescents reporting a lack of purpose and a feeling of being stuck. However, they provide little evidence on the frustration that adolescents felt during other stages of the pandemic when, although lockdown was not in force, there were severe restrictions on leisure activities, social interactions, and freedom of movement. In fact, the study by Magson et al. (2021) shows that adolescents seemed to be more worried by the restrictions imposed by the government than by the virus itself, and that this concern is related to higher levels of anxiety and depressive symptoms, and lower levels of life satisfaction As several studies show that depressive symptoms are negatively related to life satisfaction (e.g., Jovanović, 2016; Moksnes et al., 2016), it is not surprising that the COVID-19 pandemic has had an effect on both variables. Likewise, some studies show that emotional stability is related to lower levels of depression and higher levels of life satisfaction (e.g., Heisel & Flett, 2004; Hills & Argyle, 2001), so this variable may also play a relevant role.

## Comparison between early and late adolescence on the impact of the pandemic

Another issue for which there is also little evidence is whether the pandemic has affected younger and older adolescents in the same way, especially in stages of non-lockdown with the implementation of particularly restrictive measures. In principle, the impact would be expected to be higher in late adolescence than in early adolescence, because at these ages there is a greater need for freedom, autonomy and independence from parents. It should also be noted that, prior to the pandemic, more psychological distress had already been observed in late adolescence. Goldbeck et al. (2007) showed that life satisfaction tends to decrease from 11 to 16 years old, and Larson et al. (2002) showed that average affect tends to decrease from 11 to 15 years old, after which it tends to remain stable, and does not improve until later, in youth. Some studies carried out during lockdowns showed that older adolescents were more concerned than younger adolescents about the COVID-19 situation. For example, Buzzi et al. (2020) found that Italian adolescents between 17 and 19 years old reported more concerns and fears about the pandemic than the younger group of adolescents, which agrees with the results of Muñoz -Fernández & Rodríguez-Meirinhos (2021). However, as far as we know, there are no studies about such age differences in other stages of the pandemic. Nor have we found any studies about the possible role of psychological maturity in the prediction of the frustration felt by adolescents in this situation. However, several studies show that adolescents with higher levels of psychological maturity tend to suffer less psychological distress, and report higher levels of life satisfaction and emotional stability, and lower levels of depressive symptomatology and suicidal ideation (Morales-Vives & Dueñas, 2018; Morales-Vives et al., 2013). Therefore, it seems plausible that more mature adolescents would have more resources to cope with the inconvenience and constraints generated by the pandemic, resulting in less frustration and less psychological distress.

In this study we have taken as a reference the model of maturity put forward by Greenberger and Sørensen (1973), specially their concept of individual adequacy. According to this model, mature people are characterized by greater individual adjustment, which involves a willingness to fulfil their own obligations (work orientation), a willingness to take the initiative and show autonomy without allowing others to exercise excessive control (self-reliance), and good knowledge of their own characteristics and needs (identity). Several studies show that the development of identity is particularly relevant in adolescence, as it is related to emotional stability and mental health (Morales-Vives & Dueñas, 2018; Morales-Vives et al., 2013). Extraversion is another variable that is related to psychological maturity, especially the identity component of maturity (e.g., Morales-Vives et al., 2012; Morales-Vives et al., 2013; Morales-Vives & Dueñas, 2018). Extraversion reflects the tendency toward behavioral exploration (DeYoung, 2015), and this exploration may be a source of personal experience that helps to increase personal self-knowledge, giving rise to a more consolidated identity and greater psychological maturity.

## The present study

**Fig. 1:**
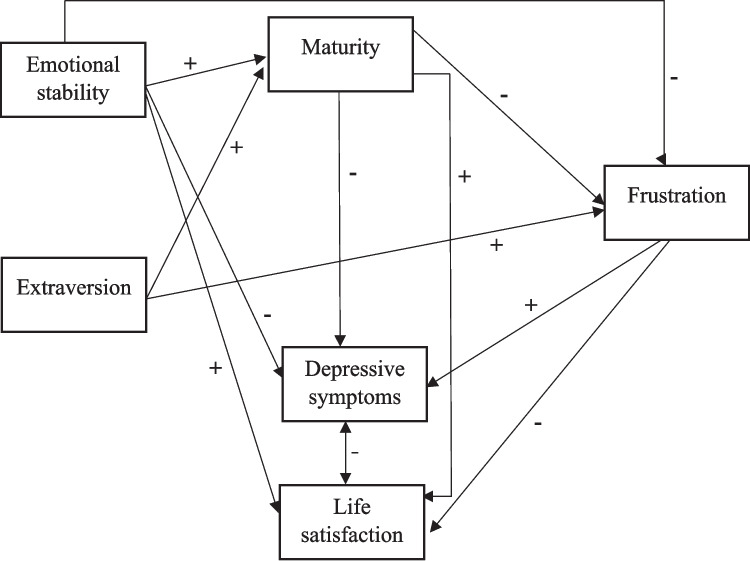
Hypothetical structural equation model

Taking all the above into account, the current study focuses on the frustration felt by adolescents because of the severe restrictions imposed by the government to control the spread of COVID-19, in a non-lockdown situation. The first goal was to determine the role of psychological maturity and the personality traits emotional stability and extraversion in the prediction of frustration. We formulated the following hypotheses for this goal:

- 1. Adolescents who are more mature and more emotionally stable will feel lower levels of frustration as a result of the pandemic.
- 2. Adolescents who are more extraverted will tend to feel more frustrated because of their greater need to socialize with others. However, we also expected that extraversion would have a negative indirect relationship with frustration because of the relationship it has with maturity (see Fig. 1), considering the previous studies mentioned above.
- 3. Higher levels of frustration will be related to higher levels of depressive symptoms and lower levels of life satisfaction.

Figure 1 shows the pattern of relationships that we expected to find between frustration and the other variables. The other relationships were defined on the basis of the results obtained in the previous studies mentioned above, which show the relationship between life satisfaction, depressive symptoms emotional stability, and psychological maturity.

The second goal of this study was to determine if adolescents between 15 and 17 years old felt higher levels of frustration than adolescents between 12 and 14 years old. The third goal was to determine if adolescents between 15 and 17 years old also reported higher levels of depressive symptoms and lower levels of life satisfaction than younger adolescents.

As the depressive symptomatology and life satisfaction detected at these different ages could be explained by mere developmental differences between these two moments of adolescence, we also aimed to determine if the levels of depressive symptomatology and life satisfaction of older adolescents during the pandemic were higher than the levels found in an equivalent sample before the pandemic. In other words, we aimed to determine whether the levels of these variables in older adolescents during the pandemic were higher than those that would be expected only because of age. If this is the case, the differences between older and younger adolescents during the pandemic could not simply have a developmental explanation, but would reflect the different impact of the pandemic on these age groups.

## Method

### Participants

To achieve the first and second goals of this study, we collected data from a sample of 774 adolescents (52.3% girls) during the months of April and May of 2021. They were recruited from two state schools in Tarragona (Spain). The age range of the participants was 12 to 17 years, with a mean of 14.4 (*SD* = 1.5). Of these, 420 (49.5% girls) were aged between 12 and 14 years, with a mean of 13.2 (*SD* = 0.8), and 354 (52.3% girls) were aged between 15 and 17 years, with a mean of 15.8 (*SD* = 0.8).

As the third goal of this study was to compare adolescents during the pandemic with adolescents before the pandemic, we compared the sample collected in 2021 with a sample collected in 2018 from two public high schools with similar characteristics to the high schools of the 2021 sample, although on that occasion data were only collected from adolescents aged 15–17 years old. In order to make the two samples comparable, the participants were paired according to sex and age, so that for each age group (15, 16 and 17 years old) there was always the same proportion of boys and girls. Therefore, each sample consists of 286 adolescents (57.0% girls) between 15 and 17 years old (*M* = 16.5, *SD* = 0.95).

So as to assess that the collected sample sizes above were sufficient for the purposes of the present research, for the three structural solutions proposed in the study (see below), a power analysis study was undertaken by using the approach proposed by Lee et al. (2012). More specifically, (a) the misspecification in *H*_*1*_ was set at a value of RMSEA = 0.08 and (b) the amount of required power was specified to be 0.80. In these conditions, the required sample sizes would be: *N* = 250 (confirmatory item factor analysis), *N* = 750 (multiple-group analysis), and *N* = 550 (pre-post analysis). Therefore, the collected sizes are acceptable in all cases. More specifically, the resulting power estimates were: β = 0.99 (confirmatory item factor analysis), β = 0.82 (multiple-group analysis), and β = 0.82 (pre-post analysis). It can be concluded that, in the three cases, there is enough power to detect small to moderate misspecifications.

### Instruments

*Beck Depression Inventory* (BDI; Beck et al., 1961)*.* We used the Spanish adaptation of this questionnaire developed by Sanz and Vázquez (1998), which has shown adequate psychometric properties. The questionnaire has 21 items on a four-point Likert scale from 0 to 3 (0 = No depressed feelings). The estimated reliability (Cronbach’s alpha) of the scores in our sample was α = 0.90.

*Satisfaction With Life Scale* (SWLS; Diener et al., 1985). We used the Spanish adaptation developed by Atienza et al. (2000). This questionnaire evaluates satisfaction with life, understood as the overall assessment that a person makes of his or her life, weighing up the good and the bad and considering personal objectives, expectations, values and interests. It is intended to measure a single dimension, and it is made up of five items with a Likert response format (1 = strongly disagree, 5 = strongly agree). The estimated reliability of the SWLS scores in our study was α = 0.83.

*Psychological Maturity Assessment Scale* (PSYMAS; Morales-Vives et al., 2012, 2013). This questionnaire assesses psychological maturity, understood as the ability to take on obligations and make responsible decisions, bearing in mind one’s own characteristics and needs and accepting the consequences of one’s own actions. It contains the scales work orientation (willingness to fulfill one’s own obligations and responsibilities), self-reliance (willingness to take the initiative, without allowing others to exercise excessive control) and identity (a clear definition and knowledge about oneself, comprising the own’s abilities, values, drives, beliefs, needs, etc.). It is a 26-item self-report instrument, and each item is answered on a five-point Likert scale: (1) Fully disagree, (5) Fully agree. This questionnaire corrects the social desirability and acquiescence biases using the procedures proposed by Ferrando et al. (2009) and Lorenzo-Seva and Ferrando (2009), and so provides bias-free scores. This questionnaire was originally developed for adolescents in the 14–18 year-old range. Because some adolescents were 12 and 13 years old in our study, we assessed the PSYMAS to determine whether it worked with the same measurement properties in this age group. More specifically, the sample was split into two age groups (above 14, *N* = 528, and below 14, *N* = 246) and the equivalence of the corresponding solutions was assessed using rotational strategies based on target rotation (e.g., Millsap & Meredith, 2007). More specifically, we assessed whether the expected theoretical solution (a) was obtained in both groups, and (b) fitted equally well in both. The results were clear: in both samples, the solution closely agreed with the theoretically expected solution in terms of both congruence (congruence coefficients above 0.85) and discrepancy (root mean discrepancy values below 0.15). The goodness of fit results were very similar in both samples and agreed with the total-sample fit results above. Overall, these results suggest that the PSYMAS retains its measurement properties in the sub-group of adolescents younger than 14 years old. In our sample, the estimated reliabilities for the factor score estimates were 0.73 for work orientation, 0.71 for self-reliance, 0.82 for identity, and 0.85 for the overall scores.

*Overall Personality Assessment Scale* (OPERAS; Vigil-Colet et al., 2013). This questionnaire assesses the Big Five personality traits, but we only used the subscales emotional stability (7 items) and extraversion (7 items). Each item is answered on a five-point Likert scale: (1) Fully disagree, (5) Fully agree. The questionnaire corrects the social desirability and acquiescence biases using the procedures described above. In our sample, the estimated reliabilities for the factor score estimates were 0.87 for emotional stability and 0.80 for extraversion.

*Frustration associated with the pandemic*. This instrument was specifically developed for the current study. It consists of seven items answered on a five-point Likert scale: (1) Fully disagree, (5) Fully agree. The process of development and its psychometric characteristics are explained below.

### Procedure

The project and the protocol of this study was approved by Research and Innovation Ethics Committee (CEIPSA) of Universitat Rovira i Virgili. This study was carried out in accordance with the recommendations of Spanish Organic Law 3/2018, of 5 December, on the Protection of Personal Data and Guarantee of Digital Rights and the Spanish Agency for Data Protection, which regulate the fundamental right to the protection of data. We sent parental permission forms to all parents of students approximately three weeks before data collection. A total of 80.2% of parents gave their consent, so the remaining 19.8% of adolescents were excluded either because their parents had not given their consent, or because they had not returned the signed form. The questionnaires were administered collectively during regular school hours, and students were guaranteed anonymity and confidentiality. They were told that participation was voluntary, and that they could refuse to participate at any moment.

To develop the instrument for assessing the frustration of adolescents during the pandemic, the researchers wrote a pool of 12 items with a Likert response scale (1 = completely disagree, 5 = Completely agree). Three external judges assessed if the content and length of the items was appropriate, and if they were easy to understand for adolescents. They suggested removing five items and making some changes to the wording of three others. After making these changes, the final instrument was made up of seven items. The items can be seen in Table 1.

**Table 1:** Pattern matrix obtained from the confirmatory factor analysis solution, and means and standard deviations of the whole sample

	Loadings	Descriptive statistics	
Item	F1	Mean	*SD*
1. In recent months, I have felt very frustrated because I haven’t been able to go out at night (bars, nightclubs, etc.) *(En los últimos meses, me he sentido muy frustrado/a por no poder disfrutar del ocio nocturno (bares, discotecas, *etc*.)*	.54	2.6	1.4
2. I feel that the pandemic is depriving me of a very important stage in my life *(Siento que la pandemia me está robando una etapa muy importante de mi vida)*	.83	3.5	1.4
3. Because of the pandemic, I feel forced to do things I don’t want to *(Por culpa de la pandemia, me veo forzado/a a hacer cosas que no quiero.)*	.43	2.8	1.3
4. Since the beginning of the pandemic, I have found it more difficult to concentrate *(Desde que empezó la pandemia, me cuesta más concentrarme)*	.47	3.0	1.4
5. When I think about what I would be doing if there were no pandemic, I feel disappointed *(Cuando pienso en lo que estaría haciendo si no existiera la pandemia, me siento decepcionado/a)*	.67	3.0	1.3
6. Generally speaking, I’ve been in a good mood over the last few months *(En general, mi estado de ánimo ha sido bueno durante los últimos meses)*	-.44	3.3	1.3
7. I feel bad because the pandemic is making me miss out on a lot of new experiences *(Me siento mal por todas las nuevas experiencias que me estoy perdiendo por culpa de la pandemia)*	.79	3.4	1.3

To determine whether this instrument had the expected unidimensional, strong factorial structure, the total 2021 sample was randomly divided into two halves. The first half was used as a calibration subsample to (a) assess the most appropriate number of factors underlying the data and (b) detect any poorly functioning items that needed to be removed. The second half was used as a validation subsample to check whether the solution attained in the first half was generalizable to different samples from the target population. As both analyses led to very similar results, we fitted a final Confirmatory Factor Analysis (CFA) solution to the overall sample.

In the second part of the study, we fitted a multiple-group (two groups) structural equation model, which included the variables we thought would be related to the frustration experienced by adolescents during the pandemic. To determine the extent to which this model was appropriate for both younger (12–14 years) and older (15–17 years) adolescents, we tested whether the condition of strong invariance was attained (see Ferrando, 1996). If it was, we next tested whether the average levels of frustration, depressive symptomatology and life satisfaction were equivalent in both groups. We should point out that a direct comparison of the mean test scores on a variable-by-variable basis would not allow univocal interpretations to be made regarding the assessment of interest, as any observed mean differences might, to a greater or lesser extent, reflect a different structure of relations in the two groups. Instead, if the structure is found to be strongly invariant, the mean group differences assessed using the model estimated intercepts can be interpreted as reflecting ‘true’ intrinsic group differences.

Finally, we tested whether the levels of depressive symptomatology and life satisfaction were equivalent in adolescents aged 15–17 before the pandemic (in 2018) and adolescents aged 15–17 during the pandemic (in 2021). The procedure was the same as that explained above: we first tested for strong measurement invariance, and, if attained, we studied mean group differences via the model-estimated intercepts.

EFAs were carried out using FACTOR 12.01.02 (Lorenzo-Seva & Ferrando, 2006). CFA and structural equation models were carried out using M*Plus* v8.7. For the remaining analyses, SPSS 28 was used.

## Results

### Factor analyses of the questionnaire about frustration related to the pandemic

We carried out an EFA with the first half of the sample to determine the most appropriate number of factors to retain and to identify the poorly functioning items. The Kaiser–Meyer–Olkin (KMO) index value was 0.82, which indicated that the correlation matrix was suitable for factor analysis. Because Parallel Analysis suggested that only one factor was underlying the data, we fitted a unidimensional solution based on the RULS criterion. The essential unidimensionality index Mean of Items Residual Absolute Value Loadings (MIREAL), reported by Ferrando and Lorenzo-Seva (2018), with an associated threshold of 0.30, had a below-threshold value of 0.23, which also suggests that the data can be treated as essentially unidimensional. The solution exhibited positive manifold, with all the items loading substantially on the common factor with estimated values between 0.46 and 0.79.

In view of these results, we again fitted a unidimensional EFA solution to the validation subsample data. The KMO index value was 0.84, which indicated that the correlation matrix was suitable for factor analysis. The overall fit indices Goodness of Fit Index (GFI) and Standardized Root Mean Square of Residuals (SRMR) were 0.99 and 0.0369, respectively. We also used the Comparative Fit Index (CFI), which involves a comparative fit with respect to the null independence model, and the Root Mean Square Error of Approximation (RMSEA), which assesses the relative fit with respect to model complexity. The CFI was 0.99 and RMSEA was 0.022. All the items had substantial loadings on the single common factor, with estimates ranging between 0.41 and 0.84. Finally, the congruence (Burt-Tucker index) and discrepancy (root mean squared difference) between the loading patterns obtained in the calibration and validation sub-samples were 0.990 and 0.058, respectively. To sum up, the unidimensional solution fitted both sub-samples quite well, the resulting structure was quite strong, and the loading estimates of this solution were virtually the same in both subsamples.

Given the results above, we fitted the unidimensional CFA solution in the overall sample, and then used the solution obtained to compute content factor score estimates for all the 774 participants. The KMO index value was now 0.84, and the goodness-of-fit results were GFI = 0.99, SRMR = 0.041, CFI = 0.98, and RMSEA = 0.066, which indicated adequacy and quite a good fit. The overall solution is in Table 1, where it can be seen that the loading estimates range between 0.43 and 0.83. The reliability of the content factor score estimates was ρ_θθ_= 0.84, which can be regarded as adequate.

### SEM: A aodel of relationships between the different variables and frustration. comparison between early and late adolescents

Table 2 shows the product-moment correlations between the intervening variables. As can be seen, frustration is negatively correlated to emotional stability, psychological maturity and life satisfaction, and positively correlated to depressive symptomatology. However, it was not correlated to extraversion.

**Table 2:** Correlation matrix between variables

	1	2	3	4	5
1. Frustration	1				
2. Extraversion	.03	1			
3. Emotional stability	-.21**	.52**	1		
4. PSYMAS overall scores	-.23**	.40**	.62**	1	
5. Life satisfaction	-.20**	.30**	.57**	.53**	1
6. Depressive symptomatology	.31**	-.35**	-.61**	-.55**	-.55**

^*^ *p* < 0.5 ** p < *.01*

A Structural Equation Model was then fitted to assess the hypothesized relationships between frustration and the other variables in the study. More specifically, emotional stability, extraversion and psychological maturity were proposed as predictors (antecedents) of pandemic frustration. Depressive symptomatology and life satisfaction were specified as consequences of pandemic frustration. Although the first-order correlation between extraversion and frustration was not significant, we considered that this outcome reflected a suppression effect. More in detail, we hypothesized that extraversion would have a direct and positive relationship with frustration, and a negative indirect relationship with frustration via maturity, and that these impacts of opposite sign would affect (suppress) the final correlation estimate. For this reason, we decided to maintain this variable in the model.

**Fig. 2:**
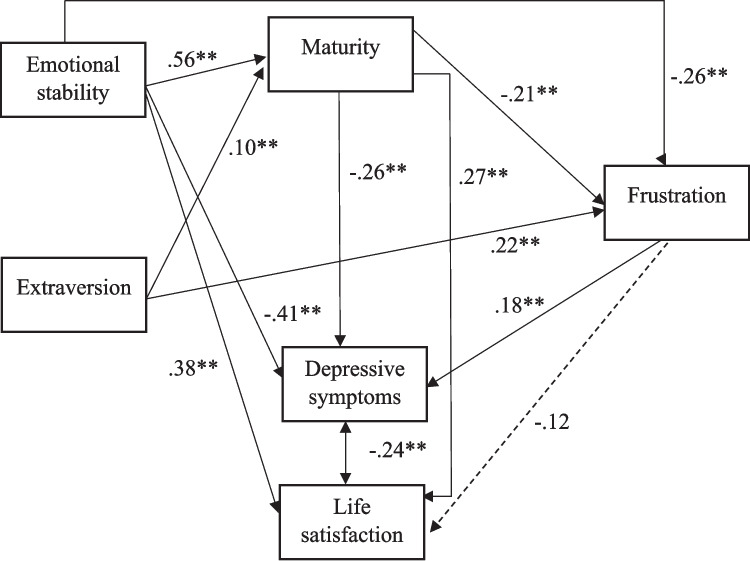
Structural equation model with standardized path estimates

First, we fitted the model in the first half of the sample to determine whether there were (a) nonsignificant paths and/or (b) variables that should be removed from the model. Then, we fitted the resulting model in the second half of the sample to determine whether this model was generalizable to different samples. Although the distributions of the variables in the model were not extreme, we fitted it using an estimation procedure (MLR) which is robust under non-normality. The goodness-of-fit results obtained in the first half of the sample were: χ^2^/*df* = 1.8, GFI = 0.99, CFI = 0.99, TLI = 0.98, SRMR = 0.011 and RMSEA = 0.045 (90% confidence interval: 0.000 and 0.120), which suggests an excellent fit. However, the path between frustration and life satisfaction was not significant, which suggests that the relationship between these variables would be indirect, through the relationship that life satisfaction has with the other variables. For this reason, we decided to remove this path, and we fitted the simplified model in the second subsample. The goodness-of-fit results were: χ^2^/*df* = 0.50, GFI = 0.99, CFI = 1.00, TLI = 1.01, SRMR = 0.008 and RMSEA = 0.000 (90% confidence interval: 0.000 and 0.065), which also suggests an excellent fit. Finally, we fitted this model in the overall sample, and the goodness-of-fit results were: χ^2^/*df* = 2.7, GFI = 0.99, CFI = 0.99, TLI = 0.98, SRMR = 0.007 and RMSEA = 0.046 (90% confidence interval: 0.000 and 0.081), which again suggests an excellent fit. The standardized regression coefficients obtained in this analysis are shown in Fig. 2. As for the compound path extraversion-maturity-frustration (the assumed indirect effect), using the standard rules of path analysis, the overall-indirect-effect point estimate was -0.021 (the result of multiplying 0.10 and -0.21). As expected, it was negative. The corresponding standard error was estimated using (a) the delta method (e.g. Raykov & Marcoulides, 2004). and (b) Bootstrap resampling based on 20,000 replications. Results (a) and (b) agreed closely and the z-statistic value was virtually the same. The compound path reached one-tailed statistical significance (z = -1.76 with delta and z = -1.77 with Bootstrap), although, clearly, the effect is small.

The model in Fig. 1 was then extended to the multiple-group case with age as a grouping variable (group 1 = 12–14 years, group 2 = 15–17 years) and with the assumption of strong measurement invariance (i.e. the covariance-based path regression parameters were the same in both groups). The fit values were: χ^2^/*df* = 1.07, GFI = 0.99, CFI = 1.00, SRMR = 0.020, and RMSEA = 0.013 (90% confidence interval: 0.000 and 0.050). The chi-square contributions were 7.41 for younger adolescents and 9.41 for older adolescents. So, the strongly invariant solution is quite tenable, and suggests that the relational structure is not different for early and late adolescence. However, although the direction and strength of the relations in the model are the same in both groups, this does not mean that the mean group levels in the intervening variables are also the same. For this reason, we tested whether the intercept estimates in frustration, life satisfaction, and depression were significantly different in the two groups. The results in Table 3 suggest that the older adolescents reported higher levels of depressive symptoms and frustration, and lower levels of life satisfaction, than early adolescents. The effect size was small for depressive symptoms and life satisfaction, and medium for frustration.

**Table 3:** Comparison between the intercept estimates in life satisfaction, depressive symptoms and frustration for early and late adolescence during the pandemic

	Early adolescence	Late adolescence	*SE*	*z*	*p*	*d*
	Intercept	Intercept				
Life Satisfaction	10.33	9.87	0.15	3.04	< .01	.11
Depressive symptoms	36.83	37.80	0.38	-2.53	< .01	.10
Frustration	50.93	52.62	0.14	-8.45	< .01	.42

*SE* = Pooled standard error*, d* = Cohen’s d effect size

### Comparison between pre-pandemic adolescents and pandemic adolescents

The comparison between pre-pandemic and pandemic mean levels in depressive symptomatology and life satisfaction was based on the same structural model described above, but without the variable frustration, which was only assessed in the sample collected in 2021 (before the pandemic, there was no point in assessing the frustration related to the pandemic). Therefore, the strongly invariant solution was fitted to the paired groups of adolescents aged 15–17 years old using year as a grouping variable (group 1 = 2018, group 2 = 2021). Data fit results were: χ^2^/*df* = 1.8, GFI = 0.99, CFI = 0.99, SRMR = 0.045, and RMSEA = 0.0055 (90% confidence interval: 0.013 and 0.92). The chi-square contributions were virtually the same: 10.65 for prepandemic adolescents and 10.08 for pandemic adolescents. So, the hypothesis of strong model invariance for prepandemic and pandemic adolescents seems quite tenable. Table 4 shows the intercept comparisons for life satisfaction and depression. The results suggest that those adolescents assessed during the pandemic (in 2021) reported higher levels of depressive symptoms than those adolescents assessed before the pandemic (in 2018), with a small effect size. However, no significant group differences in life-satisfaction were found.

**Table 4:** Comparison between the intercept estimates for 2018 and 2021 paired samples in life satisfaction and depressive symptoms

	2018 sample	2021 sample	*SE*	*z*	*p*	*d*
	Intercept	Intercept				
Life Satisfaction	6.25	6.14	0.18	0.61	> .05	-
Depressive symptoms	53.73	55.96	0.38	-5.86	< .01	.24

*SE* = Pooled standard error,* d* = Cohen’s d effect size

## Discussion

Frustration is considered a common emotional response to stress, and in the case of the circumstances surrounding the pandemic, it can be associated with situations generated by social distancing measures that may limit the attainment of such personal goals as satisfying emotional and social needs. For this reason, one aim of this research was to examine the role of maturity and two personality traits (emotional stability and extraversion) in predicting the frustration of adolescents a year after the strict period of lockdown, during a period of severe restrictions imposed by the government to control the spread of COVID-19. In line with our hypothesis, higher levels of maturity and emotional stability were associated with lower levels of frustration. We also expected that more extraverted people would report higher levels of frustration, because they tend to have a greater need to socialize, and the restrictions were a major impediment to socializing with others. The results of the Structural Equation models support this hypothesis. Our result is consistent with the finding of Wijngaards et al. (2020), which show that the lifestyle associated to stringent restrictions feels more natural to introverts than to extraverts. However, we did not find a significant product-moment correlation between extraversion and frustration. This may be explained by a suppression effect derived from the fact that extraversion has a direct and positive relationship with frustration, and also an indirect and negative relationship with this variable through maturity, which would have affected the magnitude of the first-order correlation.

As expected, the results supported the positive association between frustration and depressive symptoms. But they also suggest that frustration has an indirect relation with life satisfaction, through depressive symptoms. A plausible interpretation for these results is that those adolescents who feel more frustrated because of the strict restrictions associated to the pandemic tend to suffer more depressive symptoms, which in turn decrease their life satisfaction.

Although the proposed pattern of relationships between the variables remained invariant for early and late adolescents, the results suggest that there are significant differences in the levels of frustration, depressive symptoms and life satisfaction between these groups. As expected, older adolescents reported higher levels of frustration and depressive symptoms, and lower levels of life satisfaction. These findings are similar to those obtained by Buzzi et al. (2020) and Muñoz-Fernández and Rodríguez-Meirinhos (2021), which also show that younger adolescents tended to experience lower levels of psychological distress and less negative emotional consequences than older adolescents due to the circumstances of the pandemic. However, like many other studies that show the negative consequences of the pandemic in adolescents, these previous studies were carried out during lockdowns. The current study shows that other stages of the pandemic have also had a negative impact, particularly those stages of no lockdown but with severe restrictions. The current study also shows that the differences between these age groups are not explained only by normative developmental changes through adolescence. In fact, several studies before the pandemic showed that life satisfaction and average affect tend to decrease from 11 to 15–16 years old (e.g., Goldbeck et al., 2007; Larson et al., 2002). But the results of the current study show that older adolescents feel not only more frustration and depressive symptoms, and less life satisfaction, than younger teens, but also more frustration and depressive symptoms, and less life satisfaction, than adolescents of the same age before the pandemic. Therefore, in our opinion, these results suggest that older adolescents feel worse than younger adolescents not only for developmental reasons, but also because of the impact the pandemic is having on their lifestyles. However, this statement should be taken with caution, because in the present study there is no data from younger adolescents before the pandemic. Therefore, we could not assess if differences in wellbeing between the older and younger adolescents during the pandemic are larger than the differences before the pandemic. The results of the current study concur with those obtained by Thorisdottir et al. (2021), who also showed the impact of the pandemic on adolescents. More specifically, they assessed a sample of Icelandic adolescents between 13 and 18 years old in 2016, 2018, and 2020. The 2020 data was collected at the beginning of the pandemic, in an earlier stage than in the current study. They reported an increase in depressive symptomatology and a decrease in psychological wellbeing during the pandemic in comparison with the first assessments. Moreover, a recently published meta-analysis (Racine et al., 2021), including 29 studies on the prevalence of depressive and anxiety symptoms in young people, conclude that these problems doubled during the pandemic and are higher in older adolescents. These results agree with those reported in the present study.

In our study, no differences were found in the older adolescents’ level of life satisfaction before and during the pandemic. In contrast, Von Soest et al. (2020) found that life satisfaction in a sample of Norwegian adolescents decreased significantly when comparing the ratings reported in 2018 and those reported in a context of strict lockdown at the beginning of the pandemic. One key aspect that may explain the different result obtained in the current study is the point in time that the data was collected. While Von Soest et al. (2020) assessed their participants during the period of stricter control measures and home lockdown, our study was carried out later, in a non-lockdown situation, so the participants were not as deprived of liberty as in the other study.

The pandemic and lockdowns have had a negative impact on the psychosocial functioning and wellbeing of adolescents, disrupting their lives, and affecting them physically, emotionally, academically and socially (Branje & Morris, 2021). According to the current study, maturity and emotional stability can be considered to be protective variables that help people cope with the negative impact of post-lockdown measures adopted to reduce the spread of coronavirus, especially in populations such as adolescents who, because of the characteristics of this stage of development, are at risk of vulnerability. The daily life of young people was disrupted not only during the strict lockdown but also during the post-lockdown period. Negative feelings and distress are the result of the combination of a wide variety of factors that people had to cope with on a daily basis during the pandemic and even after periods of lockdown: for example, social isolation, stress caused by socioeconomic problems (e.g., parents’ job instability, reduction in economic activity, reduced family income), uncertainly about the future, fear of being infected or of developing chronic syndromes, etc. (Holmes et al., 2020; Imran et al., 2020).

The pandemic has led to many situations of frustration and the results of this study have revealed that this frustration negatively affects the wellbeing of adolescents. It also shows that frustration is influenced by such factors as maturity, extraversion and emotional stability, which play an important role. Furthermore, even though it is not stated as an objective, this study provides a new measure for assessing frustration during a pandemic and evidence about its factor structure in various sub-samples. This measure could be of great interest for other researchers in future pandemic situations.

Addressing the mental and physical health of adolescents, a population at risk of vulnerability, must be a priority in a public health emergency such as a pandemic (Holmes et al., 2020). The findings of this study indicate that research in these areas is needed, but not only on periods of lockdowns, because other stages of the pandemic may also be stressful for adolescents, especially older adolescents. Likewise, it is also essential to examine the long-term effects of pandemics on mental health and emotional distress in adolescents, particularly those who were at risk beforehand. Future studies should also focus on the effects on those adolescents who developed post COVID-19 syndrome, also known as "long COVID", which has had a significant impact on their daily life (Zimmermann et al., 2021).

The main limitation that should be considered when interpreting the findings is the provenance of the two samples in this study. Although we worked with paired samples, the data of the two groups (2018 and 2021) were collected through two cross-sectional studies. Therefore, we compared two different cohorts, which is a limitation that longitudinal studies do not have. This may affect the interpretability of the results to some extent, because it makes it difficult to separate developmental processes from cohort impact. The mental health during the pandemic of younger and older adolescents was also compared by a cross-sectional study. However, it should be noted that it was not possible to carry out a longitudinal study of these adolescents over the pandemic years, because the restrictions were not equally strict in all phases. These changes would have made it difficult to know whether differences in frustration over time depended on age or on the phase of the pandemic. Despite the limitations of the current study, the findings provide a robust model that explains the role of maturity, extraversion, and emotional stability in the prediction of frustration and its associated variables such as depressive symptoms. Additionally, results reveal that although the pandemic has had an impact on both groups of adolescents, the effects are greater on the older adolescents.

To sum up, the results of the current study show the negative impact that the pandemic has had on adolescents, especially older adolescents. In effect, older adolescents reported feeling more frustrated and having more depressive symptoms and lower life satisfaction than younger adolescents. Furthermore, variables such as maturity, extraversion and emotional stability play a major role in the prediction of their frustration. These results may therefore be useful for identifying vulnerable adolescents with a greater need for psychological support in a pandemic situation.

## Data Availability

The datasets generated during and/or analyzed during the current study are available from the corresponding author on reasonable request.

## Declarations

This study was performed in line with the principles of the Declaration of Helsinki. Research and Innovation Ethics Committee (CEIPSA) of Universitat Rovira i Virgili.
